# Acupuncture Modulates the Functional Connectivity of the Default Mode Network in Stroke Patients

**DOI:** 10.1155/2014/765413

**Published:** 2014-03-05

**Authors:** Yong Zhang, Kuangshi Li, Yi Ren, Fangyuan Cui, Zijing Xie, Jae-Young Shin, Zhongjian Tan, Lixin Tang, Lijun Bai, Yihuai Zou

**Affiliations:** ^1^Department of Neurology and Stroke Center, Dongzhimen Hospital Affiliated to Beijing University of Chinese Medicine, Beijing 100700, China; ^2^Department of Radiology, Dongzhimen Hospital Affiliated to Beijing University of Chinese Medicine, Beijing 100700, China; ^3^Department of Acupuncture, Dongzhimen Hospital Affiliated to Beijing University of Chinese Medicine, Beijing 100700, China; ^4^The Key Laboratory of Biomedical Information Engineering, Ministry of Education, Department of Biomedical Engineering, School of Life Science and Technology, Xi'an Jiaotong University, Xi'an 710048, China

## Abstract

Abundant evidence from previous fMRI studies on acupuncture has revealed significant modulatory effects at widespread brain regions. However, few reports on the modulation to the default mode network (DMN) of stroke patients have been investigated in the field of acupuncture. To study the modulatory effects of acupuncture on the DMN of stroke patients, eight right hemispheric infarction and stable ischemic stroke patients and ten healthy subjects were recruited to undergo resting state fMRI scanning before and after acupuncture stimulation. Functional connectivity analysis was applied with the bilateral posterior cingulate cortices chosen as the seed regions. The main finding demonstrated that the interregional interactions between the ACC and PCC especially enhanced after acupuncture at GB34 in stroke patients, compared with healthy controls. The results indicated that the possible mechanisms of the modulatory effects of acupuncture on the DMN of stroke patients could be interpreted in terms of cognitive ability and motor function recovery.

## 1. Introduction

The prevalence of stroke has increased in the past decades with the population aged and lifestyle changed [1]. Statistics from the American Heart Association state that there are more than 0.6 million people suffering from a new stroke in America every year [2]. As the leading cause of disability, stroke has greatly influenced the patients' quality of life and has left a huge burden and significant workload for their families and the whole nation [3].

Acupuncture, one of the most famous therapeutic modalities in traditional Chinese medicine, has emerged as an important modality of alternative and complementary therapeutic intervention in western medicine [4]. Recently, acupuncture has been widely used as a treatment for stroke based on a large body of clinical researches and systematic reviews which have drawn the preliminary conclusion that acupuncture is effective in stroke recovery [5–7]. However, the underlying mechanisms of these effects are not well understood.

In the last decades, noninvasive functional magnetic resonance imaging (fMRI) technique has opened a window into the brain, allowing us to investigate the central physiological function of acupuncture administration [8]. Converging evidence from fMRI studies has demonstrated that acupuncture stimulation can modulate neural activities in a wide cortico-subcortical network [9–11]. Neuroimaging studies of acupuncture have typically demonstrated extensive signal attenuations, mainly distributed in the medial temporal lobe, the posterior cingulate cortex (PCC), the medial prefrontal cortex (MPFC), and a large section of the parietal cortex [12–14]. The spatial distribution of these deactivated regions has a prominent overlap with the core regions in a “default mode” network, which is mainly present at rest and whose activities are strongly reduced during various goal-directed tasks [15, 16]. Similarly, acupuncture, not the sham condition, can also induce the increased connectivity within both the default mode network (DMN) and the sensorimotor network (SMN) [17]. It has also been reported that acupuncture at a certain acupoint, like Neiguan (PC6) and Guangming (GB37), could alter the intrinsic functional connectivity in the DMN of healthy subjects [17, 18].

Previous fMRI studies have reported specific functional connectivity changes in the DMN of stroke patients. Decreased functional connectivity in the PCC, the medial temporal lobe, and the medial prefrontal cortical areas within the DMN and reduced interregional functional connectivity between these regions have been found in stroke patients [19]. The abnormalities of the DMN functional connectivity might underlie the loss of episodic memory [19] and the occurrence of post-stroke depression and anxiety [20]. Therefore, understanding of DMN dysfunctions of stroke patients may help us comprehend the pathological mechanisms of stroke and provide additional information on brain function induced by rehabilitative interventions [21].

Given that acupuncture is effective in stroke rehabilitation and could alter the DMN in healthy subjects, we hypothesized that acupuncture would also modulate the DMN in stroke patients and this would give more interpretation of the effect of acupuncture. With healthy subjects as controls, in the current study, we tried to investigate the changes of resting-state functional connectivity in the DMN of stroke patients evoked by acupuncture at Yanglingquan (GB34), a frequently used acupoint to treat hemiplegia.

## 2. Materials and Methods

### 2.1. Subjects

A total of 8 stable ischemic stroke patients (6 males, mean age: 60.4 ± 6.6 years), diagnosed with right hemispheric corona radiate, internal capsule or basal ganglia infarction by MRI with unilateral limb disability, were recruited from Dongzhimen Hospital Affiliated to Beijing University of Chinese Medicine. All 8 patients met the following inclusion criteria: (1) first episode of stroke; (2) being between 35 and 80 years old; (3) being right-handed; (4) 2–12 weeks after the onset of stroke; (5) sufficient cognition to follow simple commands, MMSE (Mini-Mental State Examination score) > 21. Patients were excluded if they met any of the following criteria: (1) bilateral infarcts; (2) recurrent stroke; (3) any previous history of alcohol or drug abuse; (4) history of epilepsy or other neurological diseases and psychiatric disorders; (5) serious cognitive deficits; (6) being with any MRI contraindications.  Table 1 summarizes the demographic and clinical information about the stroke patients.

Another 10 right-handed normal subjects (7 males, mean age: 59.1 ± 7.8 years) were recruited to serve as healthy controls. All 10 healthy controls passed normal neurological examination and had no history of epilepsy or other neurological diseases, psychiatric disorders, or any MRI contraindications. Written informed consent was obtained from all subjects. The data was analyzed anonymously. All research procedures were approved by the ethical committee of Dongzhimen Hospital Affiliated to Beijing University of Chinese Medicine and conducted in accordance with the Declaration of Helsinki.

### 2.2. Experimental Paradigm

A multiblock paradigm is generally used in fMRI studies, which implicitly presumes the temporal intensity profiles of the certain event conforming to the “on-off” specifications. Since the acupuncture action is slow to develop and resolve [22], the temporal aspects of the BOLD response to acupuncture may violate the assumptions of the block-designed estimates. In addition, using several stimulation blocks in a short period of time, investigators may not be able to dissociate the long-lasting effects from other confounding changes, such as the effect of needle manipulation during the experiment [9, 23]. In the current study, we adopted a new experimental paradigm, namely, the nonrepeated event-related-fMRI (NRER-fMRI) design [16, 24], to investigate such prolonged effects after acupuncture administration.

The NRER-fMRI design paradigm was employed during the acupuncture stimulation, incorporating 1-minute needle manipulation, preceded by 1-minute resting epoch and followed by 8-minute resting scan (without acupuncture manipulation). Acupuncture was performed at Yanglingquan (GB34, located in the lateral aspect of the posterior knee [25]) on the left leg. The acupoint GB34, regarded as the meeting point of sinews and tendons according to the theory of Chinese medicine, is often used in the treatment of hemiplegia and motor dysfunction after stroke. Acupuncture was performed by inserting a sterile, single-use silver needle (25 mm in length and 0.30 mm in diameter) vertically into GB34 to a depth of 2-3 cm, and the stimulation consisted of rotating the needle clockwise and counterclockwise at 1 Hz with even reinforcing and reducing manipulation for 60 s. All acupuncture procedures were performed by the same experienced and licensed acupuncturist. Another 8-minute resting scan was performed before the acupuncture procedure as the baseline.

De-qi is believed to be essential to the therapeutic effectiveness of acupuncture and is often used as a signal to acupuncturists that the proper amount of needle stimulation is being performed [26, 27]. At the end of each fMRI scanning, subjects were asked to complete questionnaires to rate their experience of De-qi. Because sharp pain was considered as an inadvertent noxious stimulation, we excluded the subjects from further analysis if they experienced the sharp pain. Among all the participants, none experienced the sharp pain. Related results have been described in another paper [28].

### 2.3. Data Acquisition

fMRI images were acquired using a 3.0T MRI scanner (Siemens, Sonata, Germany). During scanning, subjects remained in the supine position with their heads immobilized by a custom-built head holder to prevent head movements. Subjects wore earplugs throughout the experiment to attenuate MRI gradient noise. Thirty-two axial slices (FOV = 225 mm × 225 mm, matrix = 64 × 64, and thickness = 3.5 mm) parallel to the AC-PC plane and covering the whole brain were obtained using a T2-weighted single-shot, gradient-recalled echo planar imaging (EPI) sequence (TR = 2000 ms, TE = 30 ms, and flip angle = 90°). Prior to the functional run, high-resolution structural information on each subject was also acquired using 3D MRI sequences with a voxel size of 1 mm^3^ for anatomical localization (TR = 1900 ms, TE = 2.52 ms, flip angle = 90°, matrix = 256 × 256, FOV = 250 mm × 250 mm, and slice thickness = 1 mm).

### 2.4. Definition of Region of Interest

The region of interest (ROI) was located in the bilateral posterior cingulate cortex (PCC, centered Montreal Neurological Institute coordinates: 0, −56, and 25), which was widely reported to be connected with other brain regions of the DMN [29] and associated with the sustained modulation effects of acupuncture [18].

### 2.5. fMRI Data Analysis

The data included the baseline resting scan and the post-acupuncture resting scan. For each resting scan, the first 10 time points were discarded to avoid the instability of the initial MRI signal. All preprocessing steps were carried out using statistical parametric mapping (SPM5, http://www.fil.ion.ucl.ac.uk/spm/). The images were first time-sliced and then realigned to correct for head motions. The image data was further processed with spatial normalization based on the MNI space and resampled at 3 mm × 3 mm × 3 mm. The functional images were spatially smoothed with an 8 mm full-width-at-half-maximum (FWHM) Gaussian kernel and then processed with a bandpass filter of 0.01–0.10 Hz. For the calculation of the functional connectivity, the correlation coefficient of each voxel was the average of the correlations (or anticorrelations) with ROI and was later normalized to *Z*-scores with Fisher's r-to-z transformation to acquire the entire brain *Z*-score map of each subject. For the group-level analyses, the functional connectivity was conducted by two-sample *t*-test and paired *t*-test using SPM5 software. The reported statistics were color-coded and mapped in Talairach space.

## 3. Results

In the resting state, the PCC showed attenuated functional connectivity with the following brain regions such as the right inferior parietal lobules, bilateral superior frontal gyrus, right middle frontal gyrus, right medial frontal gyrus, right middle temporal gyrus, right inferior temporal gyrus, and right fusiform gyrus in stroke patients compared with healthy controls. By contrast, the PCC showed increased functional connectivity with the following regions such as the bilateral precentral gyrus, bilateral postcentral gyrus, right inferior frontal gyrus, bilateral inferior parietal lobules, bilateral superior temporal gyrus, and bilateral insula in stroke patients compared with the healthy controls. Specific cluster locations are shown in Table 2.

**Figure 1:**
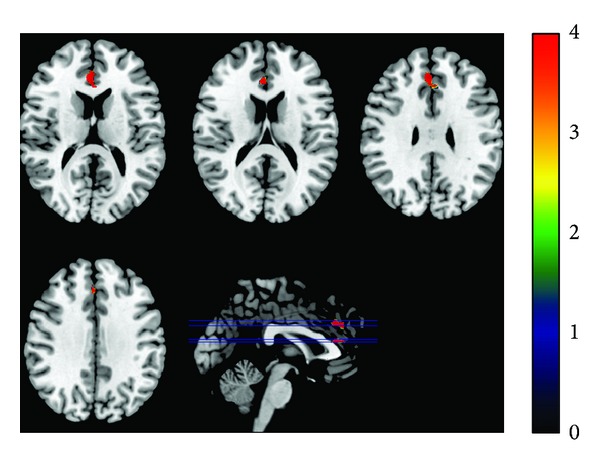
Brain regions showed increased connectivity to the PCC in stroke patients after acupuncture. Increased functional connectivity to the PCC was observed in the bilateral ACC after acupuncture in stroke patients. Results from two-sample *t*-test (df = 16, *P* < 0.01, uncorrected, corrected by Monte Carlo Simulations, and iterated 1000 times).

During the post-acupuncture resting state, the PCC showed significantly increased functional connectivity with the left middle temporal gyrus and superior temporal gyrus in healthy controls, in comparison with baseline state. For the stroke patients, acupuncture can particularly enhance the functional connectivity between the PCC and bilateral anterior cingulate cortex (ACC), while decreased functional connectivity was primarily located in the left postcentral gyrus and precentral gyrus (shown in Figure 1). Specific cluster locations are shown in Table 3.

## 4. Discussion

In the present study, resting-state fMRI scanning was applied before and after acupuncture stimulation on both stroke patients and control subjects. Resting-state functional connectivity of the DMN was analyzed, respectively, with the PCC chosen as the seed region. By doing this, we tried to test the hypothesis that acupuncture could modulate the DMN in stroke patients. The results could be divided into three continuous parts. Firstly, we detected decreased functional connectivity in the DMN of stroke patients, which is compatible to previous studies. Secondly, our results provided supportive evidence that acupuncture could modulate the DMN in healthy subjects. Finally, increased functional connectivity between the PCC and ACC was observed in the DMN of stroke patients after acupuncture at GB34, which might serve as a possible interpretation of the modulatory effects of acupuncture.

### 4.1. Resting-State Functional Connectivity before Acupuncture

We compared the resting-state functional connectivity anchored by PCC between the control subjects and stroke patients. There were a set of brain regions showing decreased or increased functional connectivity in stroke patients in comparison with the control subjects. Most of the decreased regions were involved in the frontal lobe and temporal lobe, which partly overlapped with the medial temporal lobe (MTL) and the medial prefrontal cortical (MPFC) areas within the main regions of the DMN. These results were consistent with a recent study that detected decreased functional connectivity in the PCC, MTL, and MPFC [19]. All stroke patients involved in our study were diagnosed with right hemispheric infraction. Based on our findings, most of the brain regions showed decreased functional connectivity to the PCC located in the right hemisphere which was consistent with the infraction lesions. This has confirmed the accuracy of our results to some extent. At the same time, some brain regions which were closely related to the motor and sensory functions showed increased functional connectivity to the PCC. These increases could be interpreted as compensatory reallocation or recruitment of motor and sensory functions during the functional reorganization procedures of brain networks as have been confirmed by previous fMRI studies [30–32].

### 4.2. Acupuncture Modulates the DMN in Healthy Subjects

In the current resting-state fMRI study, we observed increased functional connectivity to the PCC in the left middle temporal gyrus and the left superior temporal gyrus after acupuncture at GB34 compared with that before acupuncture in healthy subjects. Another resting-state fMRI study showed that acupuncture at Neiguan (PC6) induced increased DMN connectivity with pain, affective and memory regions including the ACC, the hippocampal formation, and the middle temporal gyrus [17]. Other resting-state fMRI studies have demonstrated that both the PCC and the anterior insula play key roles in the modulatory effects of different acupoints on the DMN in healthy subjects [16, 18, 33]. A task related fMRI study revealed that the brain regions both deactivated and activated by acupuncture at different acupoints overlapped with the DMN in healthy subjects [34]. Previous fMRI studies have drawn the conclusion that acupuncture could modulate the DMN in healthy subjects [35]. However, different modulation patterns exist among different acupoints due to the acupoint specificity [18, 34, 35], which suggested that the regions involved in fMRI studies with different acupoints may differ from each other.

### 4.3. Acupuncture Modulates the DMN of Stroke Patients

Several studies focusing on the neural effects of acupuncture on stroke patients have already been carried out [36–38]. However, there are few published studies available examining the effects of acupuncture on the DMN in stroke patients. In the current study, we applied resting-state functional connectivity to investigate the modulatory effects of acupuncture on the DMN of stroke patients with healthy subjects as control. We found that the ACC which overlap with regions underlying the DMN showed significantly increased functional connectivity with the PCC in stroke patients after acupuncture at GB34.

The ACC and PCC are considered as important components of the DMN [29]. Both PCC and ACC are regarded as core regions involved in memory and cognitive processing and their interactions with other brain networks may be important for conscious awareness [39, 40]. The DMN is an intrinsic resting-state network that regulates self-referential activity and consciousness [29] and its functional connectivity is decreased in stroke and other neurodegenerative diseases [19]. Thus, we inferred that the modulatory effects of acupuncture on the DMN of stroke patients could partly be interpreted as the recovery of cognitive ability.

A previous study focusing on the transition from rest to movement proved that the PCC and ACC served as important interaction hubs of the DMN and sensorimotor network (SMN) during movement-readiness state [41] indicating that the PCC and ACC are the overlapped key regions of the DMN and SMN. The structural and functional reorganization of the SMN after stroke has been confirmed by previous studies [42]. Previous task related fMRI studies focusing on the neurological effects of acupuncture at GB34 also induced a set of motor related brain regions involved in the SMN [43, 44]. Thus, the increased functional connectivity between the PCC and ACC induced by acupuncture at GB34 in our study could also be interpreted as the effects of the enhancement of motor recovery. We also observed decreased functional connectivity in the left precentral gyrus and the left postcentral gyrus which were underlying the brain regions of the SMN. In the present study, we propose that further studies focusing on the modulatory effects of acupuncture on the SMN, an anticorrelated network of the DMN, are still needed to give comprehensive explanations of the effects of acupuncture.

### 4.4. Limitations of This Study

However, we have to point out that the results represented here were just a preliminary exploration to the effects of acupuncture on the DMN of stroke patients. Further studies with larger sample size are still needed to confirm these results.

## 5. Conclusions

In summary, the main finding demonstrated that acupuncture at GB34 in stroke patients mainly induced enhanced functional connectivity between the ACC and PCC, in comparison with the healthy controls. We inferred that the modulatory effects of acupuncture on the DMN in stroke patients could be interpreted in terms of cognitive ability and motor function recovery. Further studies with larger sample size and focusing on the effects of acupuncture on the connection between the DMN and SMN are also needed.

## Figures and Tables

**Table 1:** Demographic and clinical information about the stroke patients.

Subject	Age (years)	Gender	Side	Lesion	MMSE	NIHSS
1	56	F	R	BG	22	14
2	64	M	R	IC	30	3
3	57	M	R	IC	27	9
4	68	M	R	CR	29	5
5	57	F	R	IC	22	8
6	58	M	R	IC	30	7
7	71	M	R	IC	24	3
8	52	M	R	BG	30	5

F: female; M: male; R: right; L: left; BG: basal ganglia; IC: internal capsule; CR: corona radiate; NIHSS: National Institute of Health Stroke Scale; MMSE: Mini-Mental State Examination.

**Table 2:** Results of the control subjects and stroke patients before acupuncture.

Regions	Side	Talairach			*t*-value	Voxels
		*X*	*Y*	*Z*		
Decreased connectivity to the PCC in stroke patients						
Inferior parietal lobule	R/IL	45	−62	47	4.17	37
Superior frontal gyrus	L/CL	−6	20	60	4.32	117
Superior frontal gyrus	R/IL	3	40	45	4.55	193
Middle frontal gyrus	R/IL	24	37	42	4.16	33
Medial frontal gyrus	R/IL	6	58	3	4.10	80
Middle temporal gyrus	R/IL	53	−66	28	4.22	28
Fusiform gyrus	R/IL	59	−10	−22	5.28	30
Increased connectivity to the PCC in stroke patients						
Precentral gyrus	R/IL	62	6	11	5.10	55
Precentral gyrus	L/CL	−48	−2	8	4.60	235
Postcentral gyrus	R/IL	62	−19	31	5.49	152
Postcentral gyrus	L/CL	−62	−5	17	4.78	162
Inferior parietal lobule	R/IL	62	−22	29	4.84	163
Inferior parietal lobule	L/CL	−53	−28	21	4.52	136
Inferior frontal gyrus	R/IL	50	5	33	7.48	62
Superior temporal gyrus	R/IL	50	14	−8	4.82	39
Superior temporal gyrus	L/CL	−56	−25	15	4.49	65
Insula	R/IL	36	−11	9	4.97	111
Insula	L/CL	−33	−14	15	4.86	171

Results from two-sample *t*-test (df = 16, *P* < 0.01, uncorrected, corrected by Monte Carlo Simulations, iterated 1000 times, and cluster size > 76 voxels). R: right; L: left; IL: ipsilateral; CL: contralateral.

**Table 3:** Results of the control subjects and stroke patients after acupuncture procedures.

Regions	Side	Talairach			*t*-value	Voxels
		*X*	*Y*	*Z*		
Increased connectivity to the PCC in control subjects						
Middle temporal gyrus	L/CL	−42	−61	9	5.84	34
Superior temporal gyrus	L/CL	−48	−46	19	6.07	29
Increased connectivity to the PCC in stroke patients						
Anterior cingulate	L/CL	−6	30	23	6.45	21
Anterior cingulate	R/IL	3	33	12	5.63	28
Decreased connectivity to the PCC in stroke patients						
Precentral gyrus	L/CL	−62	1	19	6.47	70
Postcentral gyrus	L/CL	−59	−17	15	5.39	41

Results from two-sample  *t*-test (df = 16, *P* < 0.01, uncorrected, corrected by Monte Carlo Simulations, iterated 1000 times, and cluster size > 76 voxels). R: right; L: left; IL: ipsilateral; CL: contralateral.
